# Comparative Efficacy of Chinese Herbal Injections Combined with Paclitaxel Plus Cisplatin for Non-Small-Cell Lung Cancer: A Multidimensional Bayesian Network Meta-Analysis

**DOI:** 10.1155/2020/1824536

**Published:** 2020-10-28

**Authors:** Mengwei Ni, Haojia Wang, Miaomiao Wang, Wei Zhou, Jiarui Wu, Bin Sun, Qianqian Zhang, Xiaotian Fan, Dan Zhang, Zhiwei Jing, Jingyuan Zhang, Xinkui Liu, Shuyu Liu, Ziqi Meng, Siyu Guo, Shanshan Jia, Xiaomeng Zhang, Xiaoguang Sheng

**Affiliations:** ^1^Department of Clinical Chinese Pharmacy, School of Chinese Materia Medica, Beijing University of Chinese Medicine, Beijing 100102, China; ^2^Jiangsu Jiuxu Pharmaceutical Co., Ltd., Jiangsu, China; ^3^China Academy of Chinese Medicine Sciences, Beijing 100102, China

## Abstract

**Introduction:**

Considering the limitations of pure paclitaxel plus cisplatin chemotherapy in the treatment of non-small-cell lung cancer and the extensive exploration of Chinese herbal injections, this study performed a multidimensional network meta-analysis to systematically evaluate the clinical efficacy and safety of 12 Chinese herbal injections in the treatment of non-small-cell lung cancer.

**Methods:**

Randomized controlled trials were obtained from several databases according to the eligibility criteria, and the study quality was assessed by the Cochrane risk of bias tool. Data analysis was performed by Stata 13.1 software and WinBUGS 14.0 software. Multidimensional cluster analysis was performed with the “scatterplot3d” package in *R* 3.6.1 software (PROSPERO ID: CRD42020163503).

**Results:**

A total of 58 eligible randomized controlled trials involving 4578 patients and 12 Chinese herbal injections were included. Combined with paclitaxel plus cisplatin chemotherapy, Xiaoaiping injection exhibited a better impact on the clinical effective rate than paclitaxel plus cisplatin alone. Shenqifuzheng injection was associated with a preferable response in performance status and reduced leukopenia and gastrointestinal reactions. Kangai injection was dominant in the comprehensive results of the cluster analysis.

**Conclusions:**

Chinese herbal injections combined with paclitaxel plus cisplatin chemotherapy have a certain adjuvant effect in treating non-small-cell lung cancer, but the results of this study need to be verified by more well-designed, large-sample, multicenter randomized controlled trials.

## 1. Introduction

According to the latest global cancer report, both the incidence and mortality of lung cancer ranked first among malignant tumors. Currently, the survival rate of most cancers is steadily increasing, while the 5-year relative survival rate of lung cancer is still stagnant, at 18% [1–4]. Primary bronchial lung cancer (referred to as lung cancer) is internationally divided into small-cell lung cancer and non-small-cell lung cancer (NSCLC) [5]. NSCLC accounts for approximately 85% of the total number of lung cancer cases. This value has still been increasing in the recent years, and more than 75% of patients are already in an advanced stage of lung cancer at the time of initial diagnosis and miss the opportunity for surgical treatment [6–8]. In the recent years, the treatment of NSCLC has improved, and its primary therapeutic regimens involve the combination of two drugs based on platinum drugs, such as vinorelbine plus cisplatin, paclitaxel plus cisplatin (TP), and gemcitabine plus cisplatin [9–11]. TP is a commonly used drug in chemotherapy for patients with NSCLC, and its antitumor efficacy has been demonstrated [12–16]. However, TP chemotherapy is often accompanied by the occurrence of adverse events, and how to improve clinical efficacy and reduce adverse reactions has attracted much attention [17, 18].

In China, the treatment of traditional Chinese medicine combined with chemotherapy for cancer has been widely used [19, 20]. Studies have shown that traditional Chinese medicine has beneficial effects on treating cancer, delaying cancer progression, reducing the complications caused by chemotherapy, and reducing adverse events [21–23]. Chinese herbal injections (CHIs), as an integral part of traditional Chinese medicine, play a vital role in treating cancer. Studies have shown that many components in Xiaoaiping injection have significant antitumor effects. Among them, there are dozens of C-21 steroidal saponins, which are the main anticancer components [24]. Modern pharmacological studies have indicated that these components can inhibit tumor cell proliferation, induce apoptosis, reduce the drug resistance of antitumor drugs, and improve body immunity [25–30].

Network meta-analysis (NMA) was developed from traditional meta-analysis, extending from the standard two-arm meta-analysis to indirect comparisons of multiple related but different interventions at the same time [31]. While CHIs are widely used in clinical practice, the comparison of the efficacy between multiple varieties cannot be achieved through traditional meta-analysis methods. The greatest advantage of the NMA method is that it can summarize different interventions for the treatment of a certain kind of disease, then carry out quantitative statistical analysis and rank these interventions according to the results of one outcome indicator, and finally, choose the optimal treatment plan. This NMA compares the efficacy of 12 CHIs combined with TP chemotherapy in the treatment of NSCLC by quantitatively synthesizing the evidence. This study aims to provide a reference for the clinical application of CHIs to assist in the treatment of NSCLC.

## 2. Method

This NMA was conducted according to the Preferred Reporting Items for Systematic Reviews and Meta-Analyses (PRISMA) extension statement for reporting of systematic reviews incorporating network meta-analyses of healthcare interventions [32]. A completed PRISMA checklist is included as an additional file (Checklist). A protocol, including the full methods for this review, is available from https://www.crd.york.ac.uk/prospero/display_record.php?ID=CRD42020163503.

### 2.1. Inclusion and Exclusion Criteria

Studies were considered eligible for inclusion if the following criteria were met. (1) Randomized controlled trials (RCTs) of CHIs combined with the TP chemotherapy regimen for treating NSCLC. Studies mentioning “random” were included, regardless of whether blinding methods were used. (2) All patients were diagnosed with stage III or stage IV NSCLC and had an exact pathological or cytological diagnosis. Sex and race were not limited. (3) The control group was treated with the TP chemotherapy regimen, while the observation group received CHI by intravenous drip and the TP chemotherapy regimen. There was no limitation on the dosages or treatment courses, but other adjuvant therapies (such as Chinese herbal decoction, other Chinese patent medicines, and surgical treatment) should not be used. Symptomatic treatments were given if other complications occurred. (4) The study described efficacy outcomes, such as the clinical effective rate, performance status, leukopenia, and gastrointestinal reactions [33, 34]. The primary outcome was the clinical effective rate, according to WHO's criteria for evaluating the efficacy of solid tumors, which could be divided into four levels: complete response (CR), in which visible lesions completely disappeared after >1 month; partial response (PR), in which the tumor area of a single lesion was reduced by ≥ 50% or the sum of the two largest vertical diameter products of multiple lesions was reduced by >50%; stable disease (SD), in which no significant change in condition occurred for, at least, 4 weeks and the tumor size was estimated to increase by <25% or decrease by <50%; and progressive disease (PD), in which new lesions appeared or original lesions were estimated to increase by ≥ 25%. The clinical effective rate was calculated by the following formula: clinical effective rate = (number of CR patients + number of PR patients)/total number of patients × 100%. The secondary outcomes of interest were performance status and adverse drug reactions/adverse drug events (ADRs/ADEs), including leukopenia and gastrointestinal reactions. The performance status was assessed by the Karnofsky performance status (KPS) score. KPS scores that increased by ≥ 10 points after treatment were considered to improve the performance status; KPS scores that decreased by ≥ 10 points after treatment were considered to reduce the performance status; and KPS scores that increased or decreased by < 10 points were considered stable. The performance status improvement rate = number of patients with improved performance status/total number of patients × 100%. Referring to the “Acute and Subacute Toxicity Standards of Chemotherapy Drugs” formulated by the WHO in 1981, ADRs/ADEs were divided into 5 grades. The incidence of ADRs/ADEs = number of patients with ADRs/total number of patients × 100%.

RCTs were excluded if they met the following criteria: (1) patients with other primary tumors; (2) the intervention measures were non-TP chemotherapy, or the intervention was combined with other Chinese medical treatments (such as Chinese medicine prescriptions, Chinese patent medicines, or massage), as well as Western medical treatments such as radiotherapy and surgery; (3) the administration of CHIs was nonintravenous; (4) nonclinical trials, self-controlled studies, or studies using incorrect randomization methods; (5) duplicate studies (only those with the most recent publication year, larger sample size, and more comprehensive information were retained); (6) studies with incomplete or erroneous data such as the drug name, dosage, and course of treatment; and (7) studies with inconsistent evaluation criteria and those with no related outcome indicators.

### 2.2. Search Strategy

In this NMA, RCTs were retrieved from the inception of the database to November 30, 2019, from the following databases: Embase, PubMed, the Cochrane Library, the China National Knowledge Infrastructure Database (CNKI), the Wan-fang Database, the CQVIP Database (VIP), and the China Biology Medicine disc (SinoMed). Unpublished studies and related academic organizations' websites were also researched to supplement RCTs of CHIs combined with chemotherapy for NSCLC. The search strategy, using a combination of mesh terms and free text search terms, included three parts: NSCLC, CHIs, and RCTs. Using PubMed as an example, “Non-Small-Cell Lung Carcinomas [MeSH Terms],” “Non-Small-Cell Lung Carcinoma,” “Non-Small-Cell Lung Cancer,” “Non-Small-Cell Lung Carcinoma,” “Non-Small-Cell Lung Carcinoma,” and “Non-Small-Cell Lung Cancer” were applied to identify relevant publications on NSCLC. More details about the strategy of CHIs are provided in Supplementary Materials (available here).

### 2.3. Study Selection and Data Extraction

Two researchers independently screened the titles and abstracts of potential articles to remove duplicate studies and exclude studies that definitely did not meet the inclusion criteria, and then, they cross checked the results of the included studies. Regarding repeated reports, for example, a report on the results of different periods of the same trial was included as only 1 study. Any divergences were resolved by discussion or the third reviewer. The main components of the extracted data were as follows: (1) basic information of included studies, including the title, first author's name, publication year, and source of the literature; (2) characteristics of patients, including number of patients, age, sex, KPS score before treatment, type of tumor, and clinical stage; (3) interventions, including name, dosage, course of treatment, and treatment cycle of CHIs, as well as chemotherapy drugs; (4) outcome indicators: measured data on the clinical effective rate, performance status, and ADRs/ADEs (leukopenia and gastrointestinal reactions); and (5) quality assessment items, including key factors for the type of study design and risk of bias assessment.

### 2.4. Risk of Bias Assessment

The Cochrane risk of bias tool (Cochrane Handbook for Systematic Reviews of Interventions, version 5.1.0) [35] was used to evaluate the quality of the eligible RCTs by 2 researchers. Any divergences were resolved by discussion or the third reviewer. Based on the characteristics of CHIs, we formulated quality evaluation items for the RCTs as follows: (1) whether random sequence generation and allocation concealment were mentioned (selection bias) and whether follow-up information was mentioned, including the number and reasons for withdrawals and lost follow-ups; (2) whether the blinding of the participants and personnel was mentioned (performance bias); (3) whether the blinding of the outcome assessment was mentioned (detection bias); (4) whether incomplete outcome data were mentioned (attrition bias); (5) description of eligibility criteria; (6) evaluation of ADRs/ADEs; (7) description of the statistical method; (8) funding; and (9) medical ethics.

### 2.5. Statistical Analysis

First, heterogeneity analysis was performed using the chi-square test, and the amount of heterogeneity was evaluated with the *I*^2^ statistic. It is suitable to perform NMA when slight heterogeneity exists (*I*^2^ ≤ 50%). However, if substantial heterogeneity exists (*I*^2^ > 50%), the causes of heterogeneity should first be considered. Then, we decided whether to use a random effects model after excluding clinical heterogeneity factors and, finally, performed a combined analysis. No consistency test was needed in this study because it was based on an indirect comparison comparing different varieties of CHIs combined with chemotherapy. An NMA evidence relationship diagram was performed by Stata 13.1 software (Stata Corp, College Station, TX, USA), which presents indirect comparative relationships between different interventions [36]. The statistical analysis was performed by WinBUGS 14.0 software (MRC Biostatistics Unit, Cambridge, UK). Advanced Bayesian inference with the Markov Chain Monte Carlo (MCMC) method was performed, in which the posterior probability was inferred from the prior probability and, then, estimated and extrapolated under the assumption that MCMC has reached a stable convergence state. When running the WinBUGS program, the number of iterations was set to 200 000, of which the first 10 000 iterations were used for annealing and to eliminate the effect of the initial value. The results of the indirect comparisons are expressed as the median and 95% confidence interval (95% CI) of the odds ratio (OR), which was used as the effect statistic. The relative ordering of effectiveness was obtained according to the ranking result of the median ORs. According to the surface under the cumulative ranking curve (SUCRA) value, which ranged from 0 to 1, the ranking probabilities of different interventions could be estimated; the intervention is absolutely effective when the SUCRA value is 1, while the intervention is absolutely ineffective when it is 0 [37–40]. Then, based on the SUCRA value, clustering analysis was performed on the outcome indicators, aiming to obtain the best intervention among the two clustering indicators [41]. A symmetrical comparison-adjusted funnel plot was used to assess publication bias and showed that there was no obvious publication bias in this study [42, 43].

### 2.6. Multidimensional Cluster Analysis

Multidimensional cluster analysis of the outcome indicators was performed with the “scatterplot3d” package in *R* 3.6.1 software (Mathsoft, Cambridge, USA). Using the *k*-means method to cluster these interventions, the number of clusters was adjusted according to the actual problem. The steps of *k*-means clustering were as follows: (1) All interventions were randomly divided into *k* initial classes, and the average of the outcome indicators of these *k* classes was used as the initial aggregation point. (2) Each intervention was classified into the category of the closest aggregation point; then, the aggregation point of this category was updated to the average of the current outcome indicators. All interventions were recategorized and classified. Step (2) was repeated until all interventions had been assigned, and finally, the ranking of the interventions with three outcome indicators was visualized through a three-dimensional stereogram. Different colors were applied to indicate interventions belonging to different categories.

## 3. Results

### 3.1. Literature Selection

**Figure 1:**
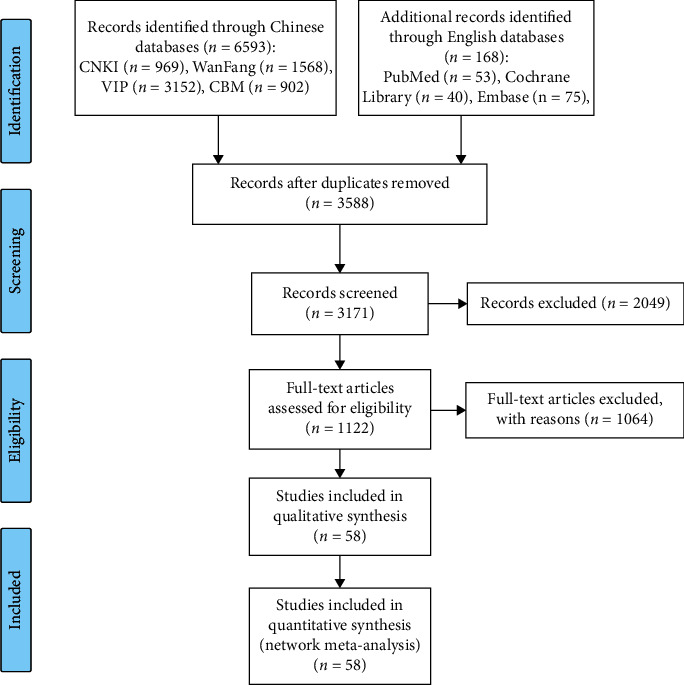
PRISMA flow diagram. (n, number of articles; CNKI, China National Knowledge Infrastructure Database; Wan-Fang, the Wan-Fang Database; VIP, the Chinese Scientific Journals Full-Text Database; and CBM, the Chinese Biomedical Literature Database).

A total of 6593 articles were retrieved in accordance with the predetermined search strategy and data collection method. After deduplication, reading the titles and abstracts, and excluding significantly the irrelevant literature, a total of 1122 RCTs of CHIs combined with the TP chemotherapy regimen in the treatment of NSCLC were collected. After reviewing the full texts of these studies, 58 RCTs [44–64], including [65–90] 12 kinds [91–101] of CHIs (Aidi injection, Huangqiduotang injection, compound Kushen injection, Delisheng injection, Huanchansu injection, Yadanziyouru injection, Kangai injection, Kanglaite injection, Shenfu injection, Shenmai injection, Shenqifuzheng injection, and Xiaoaiping injection) were included in this study. All of the studies included were Chinese literature. The study identification, screening, and inclusion process is illustrated in Figure 1.

### 3.2. Study Characteristics

**Figure 2:**
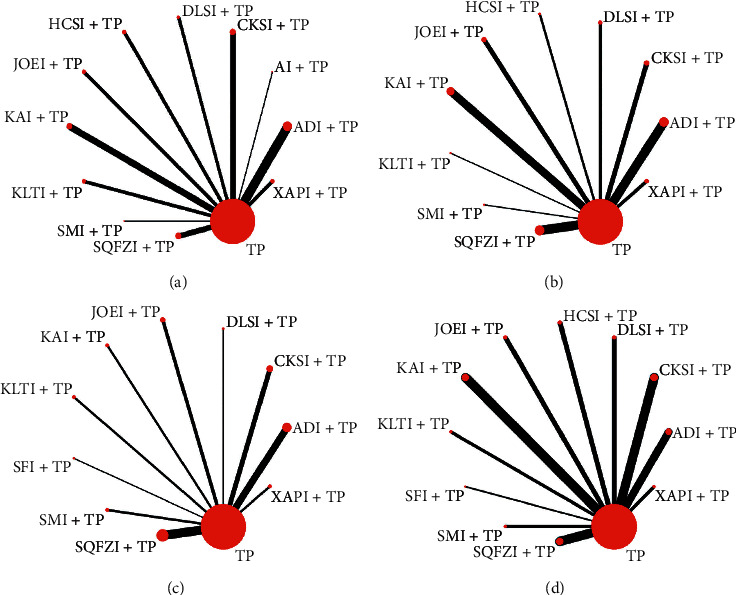
Network graph for different outcomes. (a) Clinical effective rate; (b) performance status; (c) leukopenia; and (d) gastrointestinal reactions. TP, paclitaxel plus cisplatin; XAPI, Xiaoaiping injection; ADI, Aidi injection; AI, Huangqi injection; CKSI, compound Kushen injection; DLSI, Delisheng injection; HCSI, Huachansu injection; JOEI, Yadanziyouru injection; KAI, Kangai injection; KLTI, Kanglaite injection; SMI, Shenmai injection; SQFZI, Shenqifuzheng injection; and SFI, Shenfu injection.

A total of 4578 patients were included in this study, with 2310 patients in the observation group and 2268 in the control group. All included RCTs reported the number of patients, as well as their sex, age, TNM stage, estimated survival, and KPS score before treatment. The TP chemotherapy regimen was given to the control group, and the observation group received CHI intravenously. Information on the types, doses, and treatment courses of CHIs in the observation group and information on the doses of chemotherapeutic drugs in the control group is provided in Table 1.  Figure 2 shows the network relationship of CHIs combined with TP chemotherapy in the treatment of NSCLC.

### 3.3. Quality Evaluation

**Figure 3:**
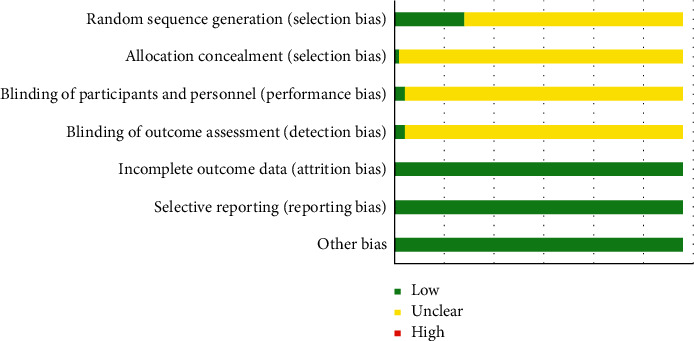
Assessment of the risk of bias.

Thirteen RCTs (22.41%) generated random sequences with the random number table method, and 1 RCT (1.72%) used the lottery method, so their selection bias due to random sequence generation was evaluated as “low risk.” The remaining RCTs that only mentioned “random” were evaluated as “unclear.” Two RCTs (3.45%) mentioned the implementation of a blinding method, and 1 RCT (1.72%) mentioned the use of a hidden random sequence, so their performance bias and detection bias were evaluated as “low risk.” Most RCTs that did not apply random sequence concealment and blindness were evaluated as “unclear.” In addition, as all included studies did not have incomplete outcomes or selective reporting, their attrition bias, reporting bias, and other bias were all evaluated as “low risk.” Specific information about the risk of bias is shown in Figure 3.

### 3.4. Clinical Effective Rate

The NMA on the clinical effective rate included 50 RCTs, 12 interventions, and 11 kinds of CHIs. The following CHIs combined with TP chemotherapy were significantly more effective than using TP chemotherapy alone: Aidi injection (OR = 0.64; 95% CI, 0.47–0.86), compound Kushen injection (OR = 0.54; 95% CI, 0.37–0.77), Kangai injection (OR = 0.45; 95% CI, 0.30–0.66), Kanglaite injection (OR = 0.49; 95% CI, 0.32–0.76), Shenmai injection (OR = 0.86; 95% CI, 0.29–2.46), and Yadanziyouru injection (OR = 0.39; 95% CI, 0.23–0.64). Based on TP chemotherapy, Xiaoaiping injection might hold greater potential for increasing the clinical effective rate than Delisheng injection (OR = 2.18; 95% CI, 1.05–4.58), and the difference between the groups was statistically significant. There were no significant differences in the clinical effective rate between the other CHIs (Table 2).

**Figure 4:**
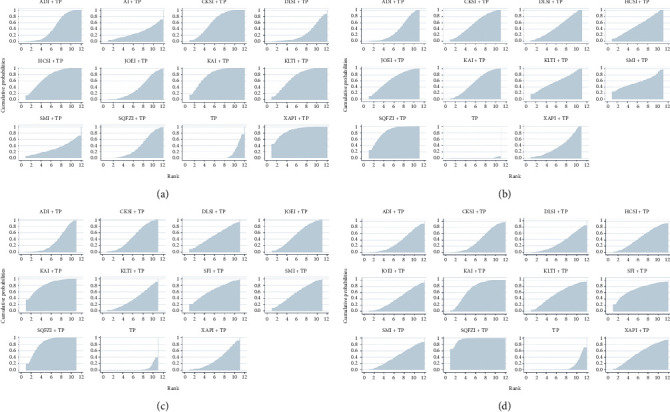
Surface under the cumulative ranking curves for all treatments. (a) Clinical effective rate; (b) performance status; (c) leukopenia; (d) gastrointestinal reactions. TP, paclitaxel plus cisplatin; XAPI, Xiaoaiping injection; ADI, Aidi injection; AI, Huangqi injection; CKSI, compound Kushen injection; DLSI, Delisheng injection; HCSI, Huachansu injection; JOEI, Yadanziyouru injection; KAI, Kangai injection; KLTI, Kanglaite injection; SMI, Shenmai injection; SQFZI, Shenqifuzheng injection; and SFI, Shenfu injection.

Based on the ranking results of improving the clinical effective rate, the relative ranking of interventions was as follows: Xiaoaiping injection (88.2%) > Kangai injection (79.2%) > Huachansu injection (73.95%) > Kanglaite injection (71.62%) > compound Kushen injection (64.13%) > Aidi injection (47.07%) > Yadanziyouru injection (39.35%) > Shenqifuzheng injection (38.13%) > Shenmai injection (31.66%) > Huangqiduotang injection (29.72%) > Delisheng injection (25.98%) > TP only (10.99%) (Figure 4).

### 3.5. Performance Status

The NMA for performance status included 41 RCTs, 11 interventions, and 10 kinds of CHIs. The following CHIs combined with TP chemotherapy were significantly more effective in improving performance status than using TP chemotherapy alone: Aidi injection (OR = 0.37; 95% CI, 0.25–0.53), compound Kushen injection (OR = 0.30; 95% CI, 0.17–0.49), Huachansu injection (OR = 0.31; 95% CI, 0.15–0.67), Yadanziyouru injection (OR = 0.27; 95% CI, 0.15–0.48), Kangai injection (OR = 0.30; 95% CI, 0.19–0.47), Kanglaite injection (OR = 0.29; 95% CI, 0.11–0.73), Shenqifuzheng injection (OR = 0.22; 95% CI, 0.15–0.34), and Xiaoaiping injection (OR = 0.38; 95% CI, 0.19–0.74) (Table 2).

Based on the ranking results of improving performance status, the relative ranking of interventions was as follows: Shenqifuzheng injection (81.82%) > Yadanziyouru injection (64.72%) > compound Kushen injection (58.32%) > Kanglaite injection (57.79%) > Shenmai injection (56.61%) > Kangai injection (55.99%) > Huachansu injection (52.59%) > Delisheng injection (47.58%) > Xiaoaiping injection (37.54%) > Aidi injection (36.61) > TP only (0.44%) (Figure 4).

### 3.6. ADRs/ADEs

#### 3.6.1. Leukopenia

The NMA for leukopenia included 31 RCTs, 11 interventions, and 10 kinds of CHIs. The following CHIs combined with TP chemotherapy were significantly more effective in reducing leukopenia than TP chemotherapy alone: compound Kushen injection (OR = 2.72; 95% CI, 1.17–6.63), Yadanziyouru injection (OR = 3.10; 95% CI, 1.09–9.06), Kangai injection (OR = 6.30; 95% CI, 1.77–23.12), and Shenqifuzheng injection (OR = 5.81; 95% CI, 3.06–11.29). Based on TP chemotherapy, Shenqifuzheng injection might hold greater potential for reducing leukopenia than Aidi injection (OR = 0.34; 95% CI, 0.13–0.92), and the difference between the groups was statistically significant. There were no significant differences in leukopenia between the other CHIs (Table 3).

Based on the ranking results of reducing leukopenia, the relative ranking of interventions was as follows: Shenqifuzheng injection (82.63%) > Kangai injection (81.24%) > Shenfu injection (64.46%) > Shenmai injection (56.24%) > Yadanziyouru injection (54.25%) > Delisheng injection (51.07%) > compound Kushen injection (48.32%) (52.59%) > Kanglaite injection (39.05%) > Xiaoaiping injection (34.74%) > Aidi injection (33.14%) > TP only (4.851%) (Figure 4).

#### 3.6.2. Gastrointestinal Reactions

The NMA for gastrointestinal reactions included 39 RCTs, 12 interventions, and 11 kinds of CHIs. The following CHIs combined with TP chemotherapy were significantly more effective in reducing gastrointestinal reactions than TP chemotherapy alone: Kangai injection (OR = 3.32; 95% CI, 1.51–7.38), and Shenqifuzheng injection (OR = 8.20; 95% CI, 3.83–18.39). Based on TP chemotherapy, Shenqifuzheng injection might hold greater potential for reducing gastrointestinal reactions than Aidi injection (OR = 0.19; 95% CI, 0.06–0.61), compound Kushen injection (OR = 0.21; 95% CI, 0.07–0.63), Delisheng injection (OR = 0.18; 95% CI, 0.05–0.66), Huachansu injection (OR = 0.24; 95% CI, 0.06–0.96), and Yadanziyouru injection (OR = 0.21; 95% CI, 0.05–0.83), and the differences between the groups were statistically significant. There were no significant differences in gastrointestinal reactions between the other CHIs (Table 3).

Based on the ranking results of reducing gastrointestinal reactions, the relative ranking of interventions was as follows: Shenqifuzheng injection (95.63%) > Kangai injection (71.31%) > Shenfu injection (70.89%) > Kanglaite injection (56.34%) > Xiaoaiping injection (53.4%) > Huachansu injection (47.53%) > Shenmai injection (43.19%) > Yadanziyouru injection (41.5%) > Fufangkushen injection (41.21%) > Aidi injection (35.37%) > Delisheng injection (32.92%) > TP only (10.71%) (Figure 4).

**Figure 5:**
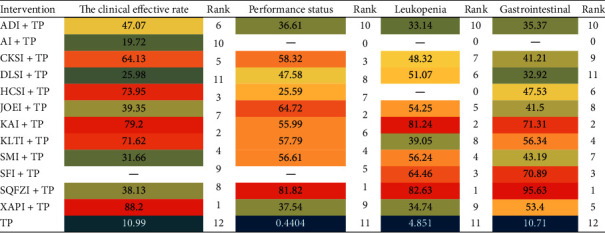
Surface under the cumulative ranking curve (SUCRA) probability results. TP, paclitaxel plus cisplatin; XAPI, Xiaoaiping injection; ADI, Aidi injection; AI, Huangqi injection; CKSI, compound Kushen injection; DLSI, Delisheng injection; HCSI, Huachansu injection; JOEI, Yadanziyouru injection; KAI, Kangai injection; KLTI, Kanglaite injection; SMI, Shenmai injection; SQFZI, Shenqifuzheng injection; and SFI, Shenfu injection.

The SUCRA heat map of all the outcome indicators is shown in Figure 5. The warmer colors represent higher SUCRA values, which indicate that this intervention is more likely to be the best intervention.

### 3.7. Cluster Analysis

**Figure 6:**
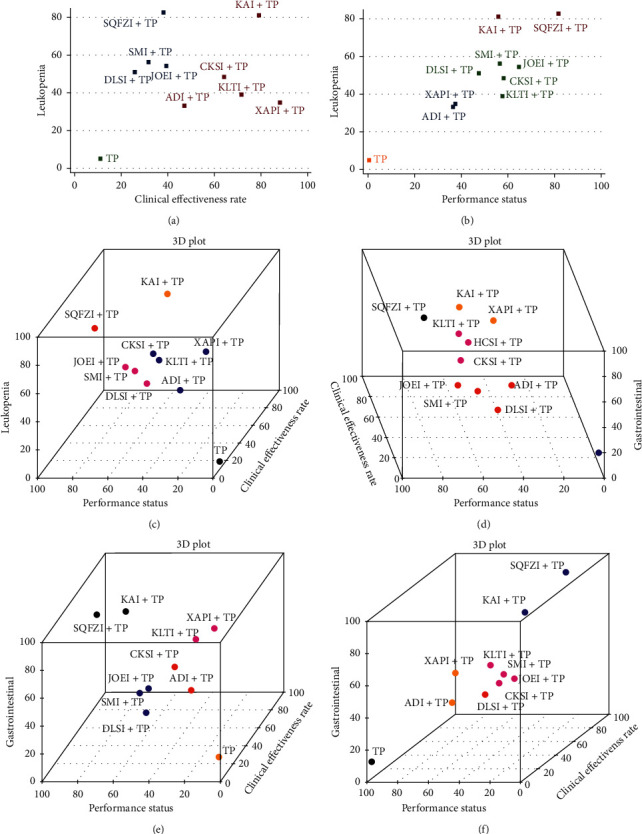
Cluster analysis plots. (a) Clinical effective rate and leukopenia; (b) performance status and leukopenia; (c) clinical effective rate, performance status, and leukopenia; (d) clinical effective rate, performance status, and gastrointestinal reactions; (e) clinical effective rate, leukopenia, and gastrointestinal reactions; and (f) performance status, leukopenia, and gastrointestinal reactions. TP, paclitaxel plus cisplatin; XAPI, Xiaoaiping injection; ADI, Aidi injection; AI, Huangqi injection; CKSI, compound Kushen injection; DLSI, Delisheng injection; HCSI, Huachansu injection; JOEI, Yadanziyouru injection; KAI, Kangai injection; KLTI, Kanglaite injection; SMI, Shenmai injection; SQFZI, Shenqifuzheng injection; and SFI, Shenfu injection.

Cluster analysis was conducted on 11 interventions that reported both the clinical effective rate and alleviation of leukopenia. Compared with other CHIs, Kangai injection combined with TP chemotherapy had the potential to be the best intervention according to the comprehensive ranking of the clinical effective rate and alleviation of leukopenia, while TP intervention only produced the worst result (Figure 6(a)). In regard to the cluster analysis of 10 interventions that reported both the improvement of performance status and alleviation of leukopenia, Kangai injection and Shenqifuzheng injection combined with TP chemotherapy had advantages in the comprehensive ranking, with TP only yielding the worst result (Figure 6(b)).

When cluster analysis was conducted with 10 interventions that reported the clinical effectiveness rate, improvement of performance status, and alleviation of leukopenia, Kangai injection combined with TP was dominant in the comprehensive ranking of all three outcome indicators, and TP only was evaluated as the worst (Figure 6(c)). In regard to the cluster analysis of 11 interventions that reported the clinical effective rate, improvement of performance status, and alleviation of gastrointestinal reactions, Shenqifuzheng injection combined with TP chemotherapy had the potential to be the best intervention according to the comprehensive ranking, while TP only was considered the worst (Figure 6(d)). The cluster analysis conducted with 10 interventions that reported the clinical effectiveness rate, alleviation of leukopenia, and alleviation of gastrointestinal reactions indicated that Kangai injection and compound Kushen injection combined with TP chemotherapy had the potential to be the best intervention (Figure 6(e)). Kangai injection and compound Kushen injection combined with TP were dominant in the comprehensive ranking of the improvement of performance status, alleviation of leukopenia, and alleviation of gastrointestinal reactions (Figure 6(f)).

### 3.8. Publication Bias

**Figure 7:**
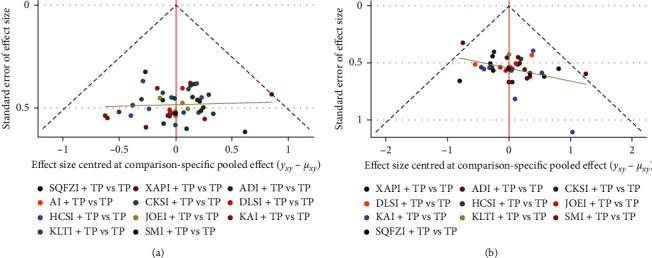
Funnel plots of publication bias. (a) Clinical effective rate; (b) performance status. TP, paclitaxel plus cisplatin; XAPI, Xiaoaiping injection; ADI, Aidi injection; AI, Huangqi injection; CKSI, compound Kushen injection; DLSI, Delisheng injection; HCSI, Huachansu injection; JOEI, Yadanziyouru injection; KAI, Kangai injection; KLTI, Kanglaite injection; SMI, Shenmai injection; and SQFZI, Shenqifuzheng injection.

Publication bias detection was performed using funnel plots for the clinical effective rate and performance status (Figure 7). There was incomplete symmetry in both funnel plots, and there were certain angles between the correction auxiliary line and the centerline, which indicated that this study has publication bias.

## 4. Discussion

This study compared the clinical efficacy and safety of 12 CHIs combined with TP in the treatment of NSCLC with the NMA method. The results are as follows. (1) In terms of the clinical effective rate, based on TP chemotherapy, Aidi injection, compound Kushen injection, Kangai injection, Kanglaite injection, Shenmai injection, and Yadanziyouru injection were significantly more effective than using TP chemotherapy alone. (2) In terms of performance status, based on TP chemotherapy, Aidi injection, compound Kushen injection, Huachansu injection, Yadanziyouru injection, Kangai injection, Kanglaite injection, Shenqifuzheng injection, and Xiaoaiping injection were significantly more effective than using TP chemotherapy alone. (3) In terms of ADRs/ADEs, based on TP chemotherapy, compound Kushen injection, Yadanziyouru injection, Kangai injection, and Shenqifuzheng injection were significantly more effective in reducing leukopenia than TP chemotherapy alone. Based on TP chemotherapy, Kangai injection and Shenqifuzheng injection were significantly more effective in reducing gastrointestinal reactions than TP chemotherapy alone. (4) In the cluster analysis, Kangai injection with TP chemotherapy was dominant in the comprehensive ranking of the clinical effective rate and alleviation of leukopenia. Based on TP chemotherapy, Kangai injection and Shenqifuzheng injection were dominant in the comprehensive ranking of the improvement of performance status and alleviation of leukopenia.

NSCLC is a common malignant tumor that has the characteristics of high mortality and insignificant early symptoms. Due to the low rate of early diagnosis and the rapid development of the disease, most patients have already reached the advanced stage of local cancer or the tumor has already metastasized at the time of diagnosis. Conventional treatments for NSCLC include surgery, radiotherapy, and chemotherapy [8, 102–104]. However, while radiation or chemotherapy kills cancer cells, it can also cause serious toxic side effects, complications, and drug resistance. Therefore, many doctors and patients with NSCLC are actively exploring other complementary and alternative therapies for cancer treatment [105, 106]. Traditional Chinese medicine has been used clinically for the treatment of cancer in China, Japan, and even other Asian countries for thousands of years. In recent years, Chinese medicine as a complementary and alternative medicine has been indicated to have unique advantages in improving the clinical symptoms of cancer patients, improving quality of life, enhancing body immunity, and extending survival. At the same time, the combined use of traditional Chinese medicine and conventional cancer treatment methods such as radiotherapy and chemotherapy also has a therapeutic effect of reducing toxicity and increasing efficacy [107]. According to relevant pharmacological studies of Yadanziyouru injection, which ranked dominantly in performance status, *Brucea javanica*, a plant in the Simaroubaceae family, has significant antimalarial and antitumor activities. In particular, fatty acid components, such as oleic acid, linoleic acid, palmitic acid, stearic acid, and arachidonic acid, have strong antitumor activity and have great killing effects on tumor cells [108–111]. The mechanism may be that they can selectively destroy the membranes and mitochondria of tumor cells, inhibit growth and DNA synthesis in tumor cells, block the cell cycle of cancer cells, improve immunity, and reverse the drug resistance to many drugs [112–115]. Shenqifuzheng injection consists of the traditional Chinese medicines Codonopsis and Astragalus. The two can be used together to promote blood circulation, eliminate blood stasis, and eliminate lumps. Modern pharmacological research shows that Codonopsis can enhance the body's immunity, increase the content of white blood cells, red blood cells, and hemoglobin, adjust gastrointestinal function, and resist fatigue. Astragalus enhances immune function and has a complete immune recovery effect on lymphocyte function in cancer patients [116–118].

At present, there are two related NMAs written in Chinese in the databases, both of which have systematically evaluated the clinical efficacy of CHIs combined with TP in the treatment of NSCLC. Compared with those two studies, the advantages of this study are reflected in the following aspects. (1) For the first time, this study used *R* software to perform a multidimensional clustering analysis on the included outcome indicators, and the results were visualized through a three-dimensional stereogram. Different interventions were compared under multiple outcome indicators. (2) This study not only updated the search time and included the largest number of studies but also conducted a comprehensive search of 22 CHIs that have been used clinically for tumor treatment at this stage. The search terms used in this study were divided into three parts: NSCLC, CHIs, and RCTs. With a search strategy as the combination of mesh terms and free text, the literature retrieved for this study was comprehensive. (3) Only slight clinical heterogeneity was found among the included studies, as strict inclusion and exclusion criteria were established; the common intervention measure of the included RCTs was TP chemotherapy, and only the WHO evaluation criteria for solid tumor efficacy were adopted as the clinical efficacy evaluation criteria. (4) This study focused on not only important indicators to measure the therapeutic effect of cancer such as the clinical effective rate and improvement in performance status but also the incidence of ADRs/ADEs. Moreover, this study also performed cluster analysis and comprehensive ranking for the above outcome indicators.

## 5. Limitations

Nevertheless, there were still limitations in this study. First, the survival rate is critical in determining and judging the effectiveness of treatment for cancer patients; however, with insufficient information to perform NMA, this study did not evaluate such long-term endpoint outcome indicators. Next, the included studies did not conduct traditional Chinese medicine dialectical analysis. The systematic review should classify the data into different syndromes to provide clearer guidance for clinical practice. In addition, most of the RCTs in this systematic review had low methodological quality, small sample sizes, and incomplete descriptions of randomization and allocation. These RCTs also lack emphasis on the implementation of blinding methods and the approval of medical ethics, which may affect the reliability of the overall research. Therefore, we recommend that RCTs be registered in advance to ensure the transparency of the trial process and improve methodological quality. In addition, RCTs should be implemented in accordance with the latest clinical diagnostic and treatment guidelines. In view of the abovementioned limitations, the results of this study need to be verified by more well-designed, large-sample, multicenter RCTs.

## 6. Conclusions

In conclusion, the use of CHIs was associated with improved treatment performance and could be beneficial for patients with NSCLC compared to using TP alone. According to multidimensional cluster analysis, Kangai injection showed the best efficacy with respect to simultaneously increasing clinical efficacy and reducing ADRs/ADEs, followed by Shenqifuzheng injection and compound Kushen injection. However, more direct comparison studies of two or more CHIs are needed to further confirm the results.

## Figures and Tables

**Table 1:** The basic characteristics of the included studies.

Study ID	Sex (M/F)	Patients (T/C)	Age	KPS	Stage	Pathological type	Treatment group interventions	Control group interventions	Course of treatment	Outcome indicators
Zhang JC, 2014	43/33	38/38	37–77	≥60	IIIb, IV	LAC, LSCC, O	ADI 60 ml + PTX 175 mg/m^2^ + DDP 20 mg/m^2^	PTX 175 mg/m^2^ + DDP 20 mg/m^2^	7–14/(21 × 2)	①②
Xun P et al., 2016	91/67	80/78	41–79/33–80	≥60	IIIC, IV	LAC, LSCC, O	ADI 100 ml + PTX 175 mg/m^2^ + DDP 75 mg/m^2^	PTX 175 mg/m^2^ + DDP 75 mg/m^2^	14/(21 × 2)	①②③④
Lin X. W, 2018	NR	34/34	NR	≥60	III, IV	NR	ADI 50 ml + PTX 175 mg/m^2^ + DDP 25 mg/m^2^	PTX 175 mg/m^2^ + DDP 25 mg/m^2^	(7/21) × (4)	①②③
Huang JQ, 2013	32/24	28/28	42–75	≥60	III, IV	LAC, LSCC	ADI 100 ml + PTX 175 mg/m^2^ + DDP 25 mg/m^2^	PTX 175 mg/m^2^ + DDP 25 mg/m^2^	(14/21)	①②③
Shi ZX et al., 2016	35/27	31/31	48–77/46–73	≥60	III, IV	LAC, LSCC, LASC	ADI 40 ml + PTX 175 mg/m^2^ + DDP 25 mg/m^2^	PTX 175 mg/m^2^ + DDP 25 mg/m^2^	(21/21) × (4)	①②③
Yang Y, 2011	30/16	23/23	41–76	>60	III, IV	LAC, LSCC, O	ADI 50 ml + PTX 175 mg/m^2^ + DDP 25 mg/m^2^	PTX 175 mg/m^2^ + DDP 25 mg/m^2^	(14/21)	①②③
Huang YS, 2013	51/33	42/42	40–72	≥60	III, IV	LAC, LSCC	ADI 80 ml + PTX 175 mg/m^2^ + DDP 80 mg/m^2^	PTX 175 mg/m^2^ + DDP 80 mg/m^2^	(14/14) × (4)	①③
Zhu D, 2012	51/33	42/42	40–72	≥60	III, IV	LAC, LSCC	ADI 80 ml + PTX 175 mg/m^2^ + DDP 80 mg/m^2^	PTX 175 mg/m^2^ + DDP 80 mg/m^2^	(14/21) × (4)	①③
Zhang HY, 2012	51/33	42/42	40–72	≥60	III, IV	LAC, LSCC	ADI 80 ml + PTX 175 mg/m^2^ + DDP 80 mg/m^2^	PTX 175 mg/m^2^ + DDP 80 mg/m^2^	(14/21) × (4)	①②③
Yang G et al., 2008	56/26	40/42	28–75/30–77	≥60	III, IV	LAC, LSCC, LCLC	ADI 50–100 ml + PTX 135 mg/m^2^ + DDP 25–30 mg/m^2^	PTX 135 mg/m^2^ + DDP 25–30 mg/m^2^	(10/21) × (2)	①②③
Dang A, 2016	45/15	30/30	32–70/30–71	NR	IIIb, IV	LAC, LSCC, LASC	AI 30 ml + PTX 135 mg/m^2^ + DDP 30 mg/m^2^	PTX 135 mg/m^2^ + DDP 30 mg/m^2^	NR	①
Pang DS et al., 2011	44/18	32/30	29–76/31–75	≥70	IIIb, IV	LAC, LSCC	CKSI 20 ml + PTX 135 mg/m^2^ + DDP 30 mg/m^2^	PTX 135 mg/m^2^ + DDP 30 mg/m^2^	14/(21 × (3–6))	①②③
Wang C et al., 2010	72/40	56/56	46–70/45–68	≥70	IIIb, IV	LAC, LSCC, LASC, O	CKSI 20 mL + PTX 130–150 mg/m^2^ + DDP 80 mg/m^2^	PTX 130–150 mg/m^2^ + DDP 80 mg/m^2^	(14/21) × (4–6)	①②③
Tian SM et al., 2018	45/35	40/40	36–71/36–78	NR	IIIb, IV	LAC, LSCC	CKSI 20 ml + PTX 135 mg/m2 + DDP 30 mg/m^2^	PTX 135 mg/m2 + DDP 30 mg/m^2^	14/(21 × 3)	①②③
Long SP and Zeng JQ, 2008	80/37	60/57	44–75/48–77	>70	III, IV	LAC, LSCC	CKSI 30 ml + PTX 135 mg/m^2^ + DDP 40 mg	PTX 135 mg/m^2^ + DDP 40 mg	21 × (2–4)	①③
Han B, 2016	60/26	43/43	35–75	>60	III, IV	LAC, LSCC, LASC	CKSI 20 ml + PTX 135 mg/m^2^ + DDP 40 mg/m^2^	PTX 135 mg/m^2^ + DDP 40 mg/m^2^	21 × 2	①②③
Liu ZL and Li YF, 2011	57/21	34/34	42–77	NR	III, IV	LAC, LSCC	CKSI 60 ml + PTX 135 mg/m^2^ + DDP 40 mg/m^2^	PTX 135 mg/m^2^ + DDP 40 mg/m^2^	21 × (2–4)	①②③
Zou ML et al., 2009	44/18	32/30	30–76/31–75	≥70	III, IV	LAC, LSCC	DLSI 30–60 ml + PTX 135 mg/m^2^ + DDP 80–100 mg/m^2^	PTX 135 mg/m^2^ + DDP 80–100 mg/m^2^	7/(21 × 2)	①②③
Wu ZW and Li GF, 2006	49/11	30/30	45–70/34–70	≥70	III, IV	LAC, LSCC	DLSI 40 ml-60 ml + DOC 80 mg/m^2^ + DDP 80–100 mg/m^2^	DOC 80 mg/m^2^ + DDP 80–100 mg/m^2^	7/(28 × 2)	①②③
Wang A and Wan, LX 2008	59/39	50/48	51/52′	≥60	III, IV	LAC, LSCC, LCLC	DLSI 40 ml + DOC 75 mg/m^2^ + DDP 25 mg/m^2^	DOC 75 mg/m^2^ + DDP 25 mg/m^2^	20/(21 × 2)	①②③
He YZ et al., 2016	64/28	42/50	58.13 ± 9.05/56.91 ± 9.47^*∗*^	NR	III, IV	NR	HCSI 20 ml + PTX 135 mg/m^2^ + DDP 20 mg/m^2^	PTX 135 mg/m^2^ + DDP 20 mg/m^2^	5/(21^*∗*^3)	①③
Wang WR et al., 2013	47/43	45/45	60–85/60–83	38–52	IIIb, IV	NR	HCSI 20 ml + PTX 135 mg·m^−2^ + DDP 75 mg·m^−2^	PTX 135 mg·m^−2^ + DDP 75 mg·m^−2^	14/(21^*∗*^3)	①②③
Yu HY et al., 2012	39/25	32/32	47–72/49–71	≥60	III, IV	LAC, LSCC, LASC	HCSI 20 mL + PTX 75 mg/m^2^ + DDP 25 mg/(m^2^·d)	PTX 75 mg/m^2^ + DDP 25 mg/(m^2^·d)	28/(21^*∗*^2)	①②③
Wang YP and Shu JH, 2009	67/53	60/60	37–75/42–77	＞70	III, IV	LAC, LSCC, LASC	HCSI 20 mL + PTX 135 mg/m^2^ + DDP 25 mg/m^2^	PTX 135 mg/m^2^ + DDP 25 mg/m^2^	21^*∗*^2	①②③
Wang L, 2015	58/22	40/40	35–75	>70	IIIa, IIIb, IV	LAC, LSCC	JOEI 30 ml + PTX 125 mg/m^2^ + DDP 75 mg/m^2^	PTX 125 mg/m^2^ + DDP 75 mg/m^2^	10/21	①②③④
Fu XJ et al., 2009	40/21	31/30	29–71	>60	IIIb, IV	LAC, LSCC, LASC, LCLC	JOEI 30 ml + TAX 135 mg/m^2^ + DDP 90 mg/m^2^	TAX 135 mg/m^2^ + DDP 90 mg/m^2^	21 × 2	①②③
Xie WB et al., 2013	50/25	45/30	30–72	>60	IIIb, IV	LAC, LSCC, LASC, LCLC	JOEI 30 ml + TAX 135 mg/m^2^ + DDP 90 mg/m^2^	TAX 135 mg/m^2^ + DDP 90 mg/m^2^	21 × 2	①②③
Liu ZW, 2010	NR	33/32	NR	>60	IIIa, IIIb, IV	LAC, LSCC	JOEI 30 ml + TAX 135 mg/m^2^ + DDP 90 mg/m^2^	TAX 135 mg/m^2^ + DDP 90 mg/m^2^	21 × 2	①②
Sun LJ, 2008	86/22	60/48	45–70/48–74	>70	III, IV	LAC, LSCC, LCLC	KAI 50 ml + PTX 120 m·g/m + DDP 50 m·g/m^2^	PTX 120 m·g/m + DDP 50 m·g/m^2^	(30 × (2–4))/((21–28) × (2–4))	①②③
Zhang MJ et al., 2009	76/44	60/60	29–75/31–71	>60	III, IV	LAC, LSCC	KAI 40 ml + PTX 135 m·g/m + DDP 30 m·g/m^2^	PTX 135 m·g/m + DDP 30 m·g/m^2^	21 × 2	①②③
Zhang JL and Yang L, 2010	45/15	30/30	52–78/51–78	>60	III, IV	LAC, LSCC, LASC	KAI 40 ml + PTX 135 m·g/m + DDP 30 m·g/m^2^	PTX 135 m·g/m + DDP 30 m g/m^2^	14/(21 × 2)	①②③
Shi L, 2011	36/22	29/29	34–69/35–70	>70	IIIb, IV	NR	KAI 30 ml + PTX 135 m·g/m + DDP 75 m g/m^2^	PTX 135 m g/m + DDP 75 m g/m^2^	21 × 2	①②③
Wu DH et al., 2009	43/13	2828	36–76	>60	IIIb, IV	LAC, LSCC, LCLC, O	KAI 50 ml + PTX 135 m·g/m + DDP 100 m·g/m^3^	PTX 135 m·g/m + DDP 100 m·g/m^3^	14/(21 × 2)	①②③
Zou CP et al., 2016	40/20	30/30	51–75/52–77	NR	III, IV	NR	KAI 60 ml + PTX 150 mg/m + DDP 100 mg/m^3^	PTX 150 mg/m + DDP 100 mg/m^3^	21	③
Huang JT, 2014	33/23	28/28	67–75	>60	III, IV	LAC, LSCC	KAI 50 ml + PTX 135–175 m·g/m + DDP 75 m·g/m^3^	PTX 135–175 mg/m + DDP 75 mg/m^3^	14/(21 × 2)	①②③
Zhang SQ et al., 2014	43/12	30/25	45–80/48–84	≥60	III, IV	LAC, LSCC, LCLC	KAI 60 ml + PTX 175 mg/m + DDP 50 mg/m^3^	PTX 175 mg/m + DDP 50 mg/m^3^	21 × 2	①②③
Zhu YZ and Zhang HT, 2010	68/32	50/50	35–72/37–73	≥60	III, IV	LAC, LSCC, LASC	KLTI 100 ml + PTX 75 mg/m^2^ + DDP 25 mg/m^2^/d	PTX 75 mg/m^2^ + DDP 25 mg/m^2^/d	21^*∗*^2	①②③
Deng XN et al., 2014	42/28	34/34	63.88 ± 1.99/64.29 ± 2.07∗	≥80	IIIb, IV	LAC, LSCC	KLTI 200 mL + PTX 85 mg/m^2^ + DDP 25 mg/m^2^	PTX 85 mg/m^2^ + DDP 25 mg/m^2^	14/(21^*∗*^2)	④
He LT et al., 2017	81/27	54/54	38–80/39–78	NR	IIIb, IV	LAC, LSCC, LASC	KLTI 100 ml + PTX 75 mg/m^2^ + DDP 75 mg/m^2^	PTX 75 mg/m^2^ + DDP 75 mg/m^2^	21^*∗*^3	①②③
Jia JN, 2018	34/28	31/31	44–72/43–74	>60	III, IV	LAC, LSCC, LASC	KLTI 200 mg + PTX 75 mg/m^2^ + DDP 25 mg/m^2^	PTX 75 mg/m^2^ + DDP 25 mg/m^2^	21^*∗*^2	①③④
Tan B et al., 2014	90/36	63/63	66/64′	≥70	IIIb, IV	LAC, LSCC, LASC	KLTI 200 ml + PTX 135 mg/m^2^ + DDP 75 mg/m^2^	PTX 135 mg/m^2^ + DDP 75 mg/m^2^	21^*∗*^2	①③
Xie CH et al., 2015	53/27	40/40	20–72/39–75	NR	III, IV	LAC, LSCC, LCLC	SFI 80 ml + PTX 75 mg/m^2^ + DDP 20 mg/m^2^	PTX 75 mg/m^2^ + DDP 20 mg/m^2^	28^*∗*^2	③
Liu YQ and Jia JW, 2011	36/24	30/30	39–7 l/37–70	≥60	IIIb, IV	NR	SMI 60 ml + PTX 75 mg/m^2^ + DDP 30 mg/m^2^	PTX 75 mg/m^2^ + DDP 30 mg/m^2^	(15/21) × 2	①②③
Yang ZJ et al., 2014	51/11	32/30	41–69/43–68	NR	IIIb, IV	LAC, LSCC, LASC	SMI 100 ml + PTX 120 mg/m^2^ + DDP 75–80 mg/m^2^	PTX 120 mg/m^2^ + DDP 75–80 mg/m^2^	(14/21) × 2	①②③
Luo SW et al., 2007	39/21	30/30	33–75/34–75	>60	IIIa, IIIb, IV	LAC, LSCC	SQFZI 250 ml + PTX 135 mg/m^2^ + DDP 30 mg/m^2^	PTX 135 mg/m^2^ + DDP 30 mg/m^2^	(10–14/21)^*∗*^2	①②③
Zhang FL, 2008	43/17	30/30	39–73/36–72	>60	IIIa, IIIb, IV	LAC, LSCC	SQFZI 250 ml + PTX 135 mg/m^2^ + DDP 30 mg/m^2^	PTX 135 mg/m^2^ + DDP 30 mg/m^2^	(10–14/21)^*∗*^2	①②③
Wang LY et al., 2009	59/21	40/40	35–67/32–65	≥60	IIIa, IIIb, IV	LAC, LSCC	SQFZI 250 ml + PTX 150 mg/m^2^ + DDP 30 mg/m^2^	PTX 150 mg/m2 + DDP 30 mg/m^2^	(10–14/21)^*∗*^2	①②③
Chen YD, 2010	36/11	24/24	50–79	≥70	IIIa, IIIb, IV	LAC, LSCC	SQFZI 250 ml + PTX 135–175 mg/m^2^ + DDP 25 mg/m^2^	PTX 135–175 mg/m^2^ + DDP 25 mg/m^2^	(10/21)^*∗*^2	①②③
Cui HZ et al., 2010	36/24	32/28	62.6/58.4∗	≥60	IIIa, IIIb, IV	LAC, LSCC, LCLC	SQFZI 250 ml + PTX 135 mg/m^2^ + DDP 25 mg/m^2^	PTX 135 mg/m^2^ + DDP 25 mg/m^2^	21^*∗*^2	①②③
Mai HY et al., 2013	62/18	40/40	57–70/58–68	>50	IIIb, IV	LAC, LSCC	SQFZI 250 ml + PTX 135 mg/m^2^ + DDP 25 mg/m^2^	PTX 135 mg/m^2^ + DDP 25 mg/m^2^	21^*∗*^2	①②③
Li DH and Yang HL, 2014	57/33	50/40	39–74/38–72	>60	IIIb, IV	LAC, LSCC, LASC	SQFZI 250 ml + PTX 210 mg/m^2^ + DDP 30 mg/m^2^	PTX 210 mg/m^2^ + DDP 30 mg/m^2^	(14/21)^*∗*^2	①②③
Yuan T, 2014	41/28	35/34	43–73	≥70	III, IV	LAC, LSCC, LASC	SQFZI 250 ml + PTX 135 mg/m^2^ + DDP 25 mg/m^2^	PTX 135 mg/m^2^ + DDP 25 mg/m^2^	21	②③
Deng Y et al., 2015	102/60	78/84	(67.2 ± 9. 8)/(66. 7 ± 10.3)^*∗*^	NR	IIIb, IV	LAC, LSCC	SQFZI 250 ml + PTX 80 mg/m^2^ + DDP 75 mg/m^2^	PTX 80 mg/m^2^ + DDP 75 mg/m^2^	21^*∗*^2	③④
Su XL, 2016	43/33	38/38	(56.2 ± 3.1)/(56.4 ± 3.2)^*∗*^	NR	IIIb, IV	NR	SQFZI 250 ml + PTX 135 mg/m^2^ + DDP 30 mg/m2	PTX 135 mg/m^2^ + DDP 30 mg/m^2^	10/(21^*∗*^4)	④
Xia GA, 2013	51/27	39/39	39–77/40–76	≥50	IIIb, IV	LAC, LSCC, LASC	XAPI 40 mL + PTX 135 mg/m^2^ + DDP 30 mg/m^2^	PTX 135 mg/m^2^ + DDP 30 mg/m^2^	(14/21) × 2	①②③
Yao J, 2016	49/56	53/52	45–73/45–75	≥60	IIIb, IV	LAC, LSCC, LASC	XAPI 20 ml + PTX 135 mg/m^2^ + DDP 20 mg/m2	PTX 135 mg/m^2^ + DDP 20 mg/m2	21 × 3	①②③
Wang WY et al., 2009	37/19	27/29	39–78/38–75	≥70	IIIb, IV	LAC, LSCC, LASC	XAPI 80 ml + PTX 150–175 mg/m^2^ + DDP 70 mg/m^2^	PTX 150–175 mg/m^2^ + DDP 70 mg/m^2^	(7/21) × 2	①②③④
Mei CR et al., 2015	38/25	30/33	39–78/37–79	≥60	IIIb, IV	LAC, LSCC, LASC, LCLC	XAPI 20 ml + PTX 135 mg/m^2^ + DDP 25 mg/m^2^	PTX 135 mg/m^2^ + DDP 25 mg/m^2^	(14/21) × 2	①②③

Note: M, male; F, female; T, treatment group; C, control group; ADI, Aidi injection; AI, Huangqi injection; CKSI, compound Kushen injection; DLSI, Delisheng injection; HCSI, Huachansu injection; JOEI, Yadanziyouru injection; KAI, Kangai injection; KLTI, Kanglaite injection; SFI, Shenfu injection; SMI, Shenmai injection; SQFZI, Shenqifuzheng injection; XAPI, Xiaoaiping injection; ①, clinical effective rate; ②, performance status; ③, leukopenia; ④, gastrointestinal reactions.

**Table 2:** Statistical results of the network meta-analysis for the clinical effective rate (lower-left quarter) and performance status (upper-right quarter) outcomes (OR/MD value, 95% CI).

ADI + TP	—	1.26 (0.66, 2.47)	1.11 (0.54, 2.26)	1.18 (0.51, 2.74)	1.36 (0.68, 2.71)	1.23 (0.69, 2.19)	1.28 (0.46, 3.57)	1.26 (0.32, 5.6)	1.66 (0.94, 2.87)	0.97 (0.46, 2.1)	0.37 (0.25, 0.53)
0.72 (0.25, 2.17)	AI + TP	—	—	—	—	—	—	—	—	—	—
1.2 (0.75, 1.89)	1.67 (0.55, 5.16)	CKSI + TP	0.88 (0.39, 1.95)	0.94 (0.36, 2.32)	1.08 (0.5, 2.33)	0.98 (0.47, 1.92)	1 (0.35, 3.04)	1 (0.24, 4.63)	1.31 (0.66, 2.58)	0.77 (0.33, 1.8)	0.3 (0.17, 0.49)
0.76 (0.41, 1.39)	1.04 (0.32, 3.58)	0.63 (0.34, 1.23)	DLSI + TP	1.06 (0.4)	1.1 (0.52, 2.37)	1.15 (0.37, 3.61)	1.14 (0.27, 5.44)	1.49 (0.72, 3.1)	0.88 (0.36, 2.18)	0.33 (0.18, 0.61)
1.34 (0.78, 2.37)	1.87 (0.58, 5.99)	1.13 (0.63, 2.03)	1.77 (0.87, 3.64)	HCSI + TP	1.16 (0.44, 3.04)	1.04 (0.43, 2.51)	1.08 (0.32, 3.81)	1.08 (0.24, 5.52)	1.41 (0.59, 3.33)	0.83 (0.31, 2.29)	0.31 (0.15, 0.67)
0.9 (0.52, 1.59)	1.25 (0.39, 4.1)	0.75 (0.42, 1.43)	1.19 (0.57, 2.46)	0.67 (0.34, 1.32)	JOEI + TP	0.9 (0.43, 1.85)	0.93 (0.31, 2.84)	0.93 (0.22, 4.4)	1.22 (0.6, 2.44)	0.72 (0.3, 1.72)	0.27 (0.15, 0.48)
1.43 (0.88, 2.34)	1.99 (0.63, 6.05)	1.18 (0.7, 2.04)	1.87 (0.94, 3.61)	1.06 (0.58, 1.9)	1.56 (0.85, 2.93)	KAI + TP	1.03 (0.37, 3.02)	1.03 (0.25, 4.73)	1.35 (0.73, 2.48)	0.79 (0.36, 1.77)	0.3 (0.19, 0.47)
1.3 (0.76, 2.18)	1.79 (0.57, 5.69)	1.09 (0.61, 1.87)	1.71 (0.86, 3.33)	0.97 (0.51, 1.8)	1.43 (0.74, 2.69)	0.92 (0.49, 1.62)	KLTI + TP	1 (0.2, 5.5)	1.31 (0.46, 3.56)	0.76 (0.23, 2.42)	0.29 (0.11, 0.73)
0.75 (0.25,2.3)	1.03 (0.22, 4.76)	0.63 (0.21, 1.9)	0.98 (0.31, 3.35)	0.55 (0.17, 1.93)	0.82 (0.24, 2.73)	0.52 (0.17, 1.7)	0.57 (0.19, 2)	SMI + TP	1.32 (0.29, 5.2)	0.77 (0.16, 3.36)	0.29 (0.07, 1.09)
0.9 (0.54, 1.47)	1.25 (0.39, 3.8)	0.75 (0.43, 1.32)	1.19 (0.59, 2.31)	0.66 (0.36, 1.3)	0.99 (0.5, 1.84)	0.64 (0.34, 1.11)	0.69 (0.39, 1.28)	1.21 (0.35, 3.74)	SQFZI + TP	0.58 (0.27, 1.3)	0.22 (0.15, 0.34)
1.66 (0.93, 2.9)	2.32 (0.69, 7.43)	1.39 (0.74, 2.54)	2.18 (1.05, 4.58)	1.23 (0.63, 2.44)	1.82 (0.92, 3.74)	1.17 (0.63, 2.18)	1.27 (0.64, 2.48)	2.21 (0.64, 7.11)	1.85 (0.93, 3.52)	XAPI + TP	0.38 (0.19, 0.74)
0.64 (0.47, 0.86)	0.89 (0.31, 2.55)	0.54 (0.37, 0.77)	0.84 (0.48, 1.45)	0.48 (0.3, 0.76)	0.71 (0.43, 1.14)	0.45 (0.3, 0.66)	0.49 (0.32, 0.76)	0.86 (0.29, 2.46)	0.71 (0.47, 1.09)	0.39 (0.23, 0.64)	TP

**Table 3:** Statistical results of the network meta-analysis for the leukopenia (lower-left quarter) and gastrointestinal reaction (upper-right quarter) outcomes (OR/MD value, 95% CI).

ADI + TP	0.89 (0.28, 2.84)	1.06 (0.26, 4.37)	0.78 (0.19, 3.31)	0.88 (0.21, 3.78)	0.47 (0.14, 1.55)	0.63 (0.13, 3.08)	0.39 (0.05, 3.15)	0.85 (0.17, 4.34)	0.19 (0.06, 0.61)	0.67 (0.14, 3.35)	1.56 (0.65, 3.8)
0.73 (0.24, 2.28)	CKSI + TP	1.2 (0.32, 4.64)	0.87 (0.22, 3.51)	1 (0.26, 4.02)	0.53 (0.18, 1.61)	0.71 (0.15, 3.27)	0.44 (0.05, 3.42)	0.96 (0.2, 4.66)	0.21 (0.07, 0.63)	0.76 (0.17, 3.57)	1.75 (0.82, 3.84)
0.68 (0.12, 4.21)	0.94 (0.15, 6.07)	DLSI + TP	0.73 (0.15, 3.53)	0.83 (0.17, 4.02)	0.44 (0.11, 1.69)	0.59 (0.1, 3.27)	0.36 (0.04, 3.25)	0.8 (0.14, 4.56)	0.18 (0.05, 0.66)	0.64 (0.11, 3.52)	1.47 (0.49, 4.36)
—	—	—	HCSI + TP	1.14 (0.23, 5.71)	0.6 (0.15, 2.41)	0.81 (0.14, 4.6)	0.5 (0.05, 4.57)	1.1 (0.19, 6.45)	0.24 (0.06, 0.96)	0.87 (0.15, 4.96)	2 (0.64, 6.26)
0.64 (0.18, 2.35)	0.88 (0.22, 3.49)	0.94 (0.13, 6.66)	—	JOEI + TP	0.53 (0.13, 2.11)	0.71 (0.12, 4.03)	0.44 (0.05, 4.01)	0.96 (0.16, 5.64)	0.21 (0.05, 0.83)	0.76 (0.13, 4.35)	1.76 (0.56, 5.5)
0.32 (0.07, 1.39)	0.43 (0.09, 2.06)	0.46 (0.06, 3.74)	—	0.49 (0.09, 2.59)	KAI + TP	1.35 (0.28, 6.23)	0.82 (0.1, 6.49)	1.82 (0.38, 8.75)	0.4 (0.13, 1.22)	1.43 (0.3, 6.8)	3.32 (1.51, 7.38)
0.91 (0.21, 4.37)	1.25 (0.26, 6.39)	1.33 (0.16, 11.47)	—	1.42 (0.26, 7.97)	2.89 (0.46, 19.11)	KLTI + TP	0.61 (0.06, 6.44)	1.36 (0.21, 9.32)	0.3 (0.06, 1.41)	1.07 (0.17, 7.2)	2.47 (0.67, 9.67)
0.47 (0.07, 3.17)	0.65 (0.09, 4.59)	0.7 (0.06, 7.61)	—	0.74 (0.1, 5.67)	1.51 (0.17, 13.11)	0.52 (0.06, 4.51)	SFI + TP	2.21 (0.22, 23.34)	0.49 (0.06, 3.88)	1.75 (0.17, 18.14)	4.03 (0.6, 27.9)
0.6 (0.13, 2.87)	0.83 (0.16, 4.23)	0.89 (0.1, 7.5)	—	0.95 (0.16, 5.34)	1.92 (0.29, 12.5)	0.67 (0.09, 4.42)	1.28 (0.14, 11.56)	SMI + TP	0.22 (0.05, 1.05)	0.79 (0.12, 5.26)	1.82 (0.47, 7.11)
0.34 (0.13, 0.92)	0.47 (0.16, 1.4)	0.5 (0.08, 2.96)	—	0.53 (0.15, 1.84)	1.09 (0.26, 4.62)	0.37 (0.08, 1.63)	0.72 (0.11, 4.63)	0.56 (0.12, 2.67)	SQFZI + TP	3.55 (0.77, 16.94)	8.2 (3.83, 18.39)
1 (0.25, 4.09)	1.37 (0.31, 6.07)	1.47 (0.19, 11.21)	—	1.56 (0.31, 7.68)	3.17 (0.54, 18.17)	1.1 (0.18, 6.42)	2.11 (0.25, 17.55)	1.65 (0.27, 10.47)	2.93 (0.74, 11.38)	XAPI + TP	2.31 (0.61, 8.77)
1.98 (0.99, 4.2)	2.72 (1.17, 6.63)	2.92 (0.56, 15.27)	—	3.1 (1.09, 9.06)	6.3 (1.77, 23.12)	2.18 (0.56, 8.21)	4.19 (0.74, 24.04)	3.27 (0.84, 13.65)	5.81 (3.06, 11.29)	1.99 (0.61, 6.69)	TP

## Data Availability

The datasets we generated during the analysis are not publicly available because the analysis process is the core of the results and cannot be made public. Requests to access the datasets of included studies can be met by checking out them in databases.

## Abbreviations

95% CI: 95% confidence interval
ADRs/ADEs: Adverse drug reactions/adverse drug events
CHIs: Chinese herbal injections
CR: Complete response
KPS: Karnofsky performance status
NMA: Network meta-analysis
NSCLC: Non-small-cell lung cancer
OR: Odds ratio
PD: Progressive disease
PR: Partial response
RCTs: Randomized controlled trials
SD: Stable disease
SUCRA: Surface under the cumulative ranking curve
TP: Paclitaxel plus cisplatin.

## Supplementary Materials

Supplementary Materials This file contains three parts, which includes items regarding the PRISMA checklist for network meta-analysis and corresponding pages of this study, the search strategy of traditional Chinese medicine injections in the PubMed database, and details about the product information of CHIs.
