# Supplementary material for: Comparative Efficacy of Chinese Herbal Injections Combined with Paclitaxel Plus Cisplatin for Non-Small-Cell Lung Cancer: A Multidimensional Bayesian Network Meta-Analysis
Source: Evid Based Complement Alternat Med. 2020 Oct 28;2020:1824536. doi: 10.1155/2020/1824536 (PMC7644304; doi:10.1155/2020/1824536)
Supplement: Supplementary Materials — This file contains three parts, which includes items regarding the PRISMA checklist for network meta-analysis and corresponding pages of this study, the search strategy of traditional Chinese medicine injections in the PubMed database, and details about the product information of CHIs. [file 1824536.f1.doc]

| **Section/Topic** | **#** | **Checklist Item** | **Reported on Page #** |
| --- | --- | --- | --- |
| **TITLE** | | | |
| Title | 1 | Identify the report as a systematic review, meta-analysis, or both. | 1 |
| **ABSTRACT** | | | |
| Structured summary | 2 | Provide a structured summary including, as applicable: background; objectives; data sources; study eligibility criteria, participants, and interventions; study appraisal and synthesis methods; results; limitations; conclusions and implications of key findings; systematic review registration number. | 2 |
| **INTRODUCTION** | | | |
| Rationale | 3 | Describe the rationale for the review in the context of what is already known. | 3 |
| Objectives | 4 | Provide an explicit statement of questions being addressed with reference to participants, interventions, comparisons, outcomes, and study design (PICOS). | 3-4 |
| **METHODS** | | | |
| Protocol and registration | 5 | Indicate if a review protocol exists, if and where it can be accessed (e.g., Web address), and, if available, provide registration information including registration number. | / |
| Eligibility criteria | 6 | Specify study characteristics (e.g., PICOS, length of follow-up) and report characteristics (e.g., years considered, language, publication status) used as criteria for eligibility, giving rationale. | 4-6 |
| Information sources | 7 | Describe all information sources (e.g., databases with dates of coverage, contact with study authors to identify additional studies) in the search and date last searched. | 6 |
| Search | 8 | Present full electronic search strategy for at least one database, including any limits used, such that it could be repeated. | 6 |
| Study selection | 9 | State the process for selecting studies (i.e., screening, eligibility, included in systematic review, and, if applicable, included in the meta-analysis). | 6-7 |
| Data collection process | 10 | Describe method of data extraction from reports (e.g., piloted forms, independently, in duplicate) and any processes for obtaining and confirming data from investigators. | 6-7 |
| Data items | 11 | List and define all variables for which data were sought (e.g., PICOS, funding sources) and any assumptions and simplifications made. | 7-8 |
| Risk of bias in individual studies | 12 | Describe methods used for assessing risk of bias of individual studies (including specification of whether this was done at the study or outcome level), and how this information is to be used in any data synthesis. | 7 |
| Summary measures | 13 | State the principal summary measures (e.g., risk ratio, difference in means). | 7-8 |
| Synthesis of results | 14 | Describe the methods of handling data and combining results of studies, if done, including measures of consistency (e.g., I^2^) for each meta-analysis. | 7-8 |

Page 1 of 2

| **Section/Topic** | **#** | **Checklist Item** | **Reported on Page #** |
| --- | --- | --- | --- |
| Risk of bias across studies | 15 | Specify any assessment of risk of bias that may affect the cumulative evidence (e.g., publication bias, selective reporting within studies). | 7-8 |
| Additional analyses | 16 | Describe methods of additional analyses (e.g., sensitivity or subgroup analyses, meta-regression), if done, indicating which were pre-specified. | 8-9 |
| **RESULTS** | | | |
| Study selection | 17 | Give numbers of studies screened, assessed for eligibility, and included in the review, with reasons for exclusions at each stage, ideally with a flow diagram. | 9-10 |
| Study characteristics | 18 | For each study, present characteristics for which data were extracted (e.g., study size, PICOS, follow-up period) and provide the citations. | 11 |
| Risk of bias within studies | 19 | Present data on risk of bias of each study and, if available, any outcome level assessment (see item 12). | 18 |
| Results of individual studies | 20 | For all outcomes considered (benefits or harms), present, for each study: (a) simple summary data for each intervention group (b) effect estimates and confidence intervals, ideally with a forest plot. | 18-24 |
| Synthesis of results | 21 | Present the main results of the review. If meta-analyses done, include for each, confidence intervals and measures of consistency. | 27 |
| Risk of bias across studies | 22 | Present results of any assessment of risk of bias across studies (see Item 15). | 27 |
| Additional analysis | 23 | Give results of additional analyses, if done (e.g., sensitivity or subgroup analyses, meta-regression [see Item 16]). | 24-26 |
| **DISCUSSION** | | | |
| Summary of evidence | 24 | Summarize the main findings including the strength of evidence for each main outcome; consider their relevance to key groups (e.g., healthcare providers, users, and policy makers). | 27-29 |
| Limitations | 25 | Discuss limitations at study and outcome level (e.g., risk of bias), and at review-level (e.g., incomplete retrieval of identified research, reporting bias). | 30 |
| Conclusions | 26 | Provide a general interpretation of the results in the context of other evidence, and implications for future research. | 30 |
| **FUNDING** | | | |
| Funding | 27 | Describe sources of funding for the systematic review and other support (e.g., supply of data); role of funders for the systematic review. | 31-32 |

*From:* Moher D, Liberati A, Tetzlaff J, Altman DG, The PRISMA Group (2009). Preferred Reporting Items for Systematic Reviews and Meta-Analyses: The PRISMA Statement. PLoS Med 6(6): e1000097. doi:10.1371/journal.pmed1000097

For more information, visit: **www.prisma-statement.org**.

Page 2 of 2

The search strategy used in the English databases

**1. Search strategy of Pubmed**

#1 Non-Small-Cell Lung Carcinomas [MeSH Terms]

#2 Non-Small-Cell Lung Carcinoma [Title/Abstract]

#3 Nonsmall Cell Lung Cancer [Title/Abstract]

#4 Non Small Cell Lung Carcinoma [Title/Abstract]

#5 Non-Small Cell Lung Carcinoma [Title/Abstract]

#6 Non-Small Cell Lung Cancer [Title/Abstract]

#7 #1 OR #2 OR #3 OR #4 OR #5 OR #6

#8 Shenmai[Title/Abstract]

#9 Delisheng[Title/Abstract]

#10 Astragalus[Title/Abstract]

#11 Huangqi[Title/Abstract]

#12 Shengmai[Title/Abstract]

#13 Chansu[Title/Abstract]

#14 Toad venom[Title/Abstract]

#15 Huachansu[Title/Abstract]

#16 Cinobufacini[Title/Abstract]

#17 Shenfu[Title/Abstract]

#18 Yadanziyouru[Title/Abstract]

#19 Javanica oil emulsion[Title/Abstract]

#20 Xiaoaiping[Title/Abstract]

#21 Marsdenia Tenacissima[Title/Abstract]

#22 Kangai[Title/Abstract]

#23 Aidi[Title/Abstract]

#24 Shenqifuzheng[Title/Abstract]

#25 Kanglaite[Title/Abstract]

#26 Compound matrine[Title/Abstract]

#27 Fufangkushen[Title/Abstract]

#28 Compound Kushen[Title/Abstract]

#29 #8 OR #9 OR #10 OR #11 OR #12 OR #13 OR #14 OR #15 OR #16 OR #17 OR #18 OR #19 OR #20 OR #21 OR #22 OR #23 OR #24 OR #25 OR #26 OR #27 OR #28

#30 randomized controlled trial[Publication Type]

#31 controlled clinical trial[Publication Type]

#32 randomized[Title/Abstract]

#33 placebo[Title/Abstract]

#34 randomly[Title/Abstract]

#35 trial[Title/Abstract]

#36 groups[Title/Abstract]

#37 "drug therapy" [Subheading]

#38 #30 OR #31 OR #32 OR #33 OR # 34 #36 OR #37 OR #38

#39 #7 AND #29 AND #38

**2. Search strategy of Embase**

#1 random*

#2 placebo*

#3 doubl* adj blind*

#4 singl* adj blind*

#5 assign*

#6 allocat*

#7 “double-blind procedure”/exp

#8 ”randomized controlled trial”/exp

#9”single-blind procedure”/exp

#10 #1 or #2 or #3 or #4 or #5 or #6 or #7 or #8 or #9

#11 '' Non-Small-Cell Lung Carcinomas '/exp

#12 Non-Small-Cell Lung Carcinoma

#13 Nonsmall Cell Lung Cancer

#14 Non Small Cell Lung Carcinoma

#15 Non-Small Cell Lung Carcinoma

#16 Non-Small Cell Lung Cancer

#17 #11 OR #12 OR #13 OR #14 OR #15 OR #16

#18 shenmai

#19 delisheng

#20 huangqi

#21 astragalus

#22 shengmai

#23 chansu

#24 toad AND venom

#25 huachansu

#26 cinobufacini

#27 shenfu

#28 yadanziyouru

#29 javanica AND oil AND emulsion

#30 xiaoaiping

#31 marsdenia AND tenacissima

#32 kangai

#33 aidi

#34 shenqifuzheng

#35 kanglaite

#36 compound AND matrine

#37 compound AND kushen

#38 fufangkushen

#39 #18 OR #19 OR #20 OR #21 OR #22 OR #23 OR #24 OR #25 OR #26 OR #27 OR #28 OR #29 OR #30 OR #31 OR #32 OR #33 OR #34 OR #35 OR #36 OR #37 OR #38

#46 #10 AND #17 AND #39

**3. Search strategy of Cochrane Library**

#1 random*

#2 shenmai: ti,ab,kw

#3 delisheng: ti,ab,kw

#4 huangqi: ti,ab,kw

#5 astragalus: ti,ab,kw

#6 shengmai: ti,ab,kw

#7 chansu: ti,ab,kw

#8 toad AND venom: ti,ab,kw

#9 huachansu: ti,ab,kw

#10 cinobufacini: ti,ab,kw

#11 shenfu: ti,ab,kw

#12 yadanziyouru: ti,ab,kw

#13 javanica AND oil AND emulsion : ti,ab,kw

#14 xiaoaiping: ti,ab,kw

#15 marsdenia AND tenacissima: ti,ab,kw

#16 kangai: ti,ab,kw

#17 aidi: ti,ab,kw

#18 shenqifuzheng: ti,ab,kw

#19 kanglaite: ti,ab,kw

#20 compound AND matrine: ti,ab,kw

#21 compound AND kushen: ti,ab,kw

#22 fufangkushen: ti,ab,kw

#23 #2 or #3 or #4 or #5 or #6 or #7 or #8 or #9 #10 #11 OR #12 OR #13 OR #14 OR #15 OR #16 #17 #18 OR #19 OR #20 OR #21 OR #22

#24 Non-Small-Cell Lung Carcinomas: MeSH descriptor

#25 #1 AND #23 AND #24

Information about the included injections

| Name of injection | Source | Species / Raw materials | Botanical plant names | Component ingredients to be measured | Therapeutic claims in TCM | Indications | Adverse drug reactions | Quality control reported? (Y/N) | Chemical analysis reported? (Y/N) |
| --- | --- | --- | --- | --- | --- | --- | --- | --- | --- |
| Aidi injection | Guizhou Yibai Pharmaceutical Co., Ltd. | *Mylabris* 1.5g (animal drug), *Ginseng Radix Et Rhizoma* 50g, *Astmgali Radix* 100g, *Acanthopanacis Senticosi Radix Et Rhizoma* Seu Caulis 150g | *Panax ginseng C.A.Mey.;* *Astragalus mongholicus Bunge.*; *Eleutherococcus senticosus (Rupr. & Maxim.) Maxim.* | Ginsenoside Re, ＞2mg/10mL; Cantharidin, 0.008-0.030mg/10mL | Clearing heat and detoxifying, eliminating blood stasis | Primary liver cancer, lung cancer, rectal cancer, malignant lymphoma, gynecological malignant tumors, etc. | Patients occasionally have flushing, urticaria, fever, and very few patients have palpitations, chest tightness, nausea and other reactions. | Y-National Pharmaceutical Standard Z52020236; Standard number: WS_3_-B-3809-99-2002 | N |
| Astragalus injection | Harbin Zhenbao Pharmaceutical Co., Ltd.;  Shanghai Fuda Pharmaceutical Co., Ltd.;  Chengdu DIAO Jiuhong Pharmaceutical Factory;  Zhengdaqingchunbao Pharmaceutical Co., Ltd.;  Shanghai Hefeng Pharmaceutical Co., Ltd.;  Dali Pharmaceutical Co., Ltd.;  WuxiJiMinKeXinShanHePharmaceutical Co. Ltd.;  Shineway Pharmaceutical Group Ltd.;  Shanghai Xinya Pharmaceutical Gaoyou Co., Ltd.;  Shanxi Jinxin Shuanghe Pharmaceutical Co., Ltd.;  Heilongjiangzbd Pharmaceutical Co.,Ltd.;  Zhejiang Jiuxu Pharmaceutical Co., Ltd.;  HARBIN SANCTITY BIOLOGICAL PHARMACEUTICAL CO.,LTD. | *Astmgali Radix* 20g | *Astragalus mongholicus Bunge.* | Astragaloside IV, ＞0.08mg/mL | Benefit Qi and nourish the Yuan, support the right and remove evil, nourish the heart and tonify the pulse, strengthen the spleen and dampness | Viral myocarditis, cardiac insufficiency and hepatitis,adjuvant therapy for malignant tumors | Allergic reactions; dyspnea, asthma, chest tightness; phlebitis, atrial fibrillation; liver damage, vomiting, diarrhea; headache, kidney damage; rare hemolytic anemia | Y-Standard number: WS_3_-B-3335-98 | N |
| Chansu injection | Jiangsu Pujin Pharmaceutical Co., Ltd.;  Anhui Koyo Pharmaceutical Co.,Ltd. | *Bufonis Venenum* 2g (animal drug) | *-* | Serotonin ,＞18.0μg/mL | Detoxification and pain relief, enlightenment | Acute and chronic suppurative infections; anti-tumor, anti-radiation aids | Pain, allergic reactions, poor intravenous drip | Y-National Pharmaceutical Standard Z32020694, Z34020603; Standard number: WS_3_-B-3354-98 | N |
| Compound kushen injection | Shanxi Zhendong Pharmaceutical Co., Ltd. | *Radix Sophorae Flavescentis* 1400g, Baituling 600g | *Sophora flavescens Aiton*; *Smilax china L.* | Matrine, ＞18mg/mL | Clearing away heat and dampness, cooling blood and detoxifying, loosening pain | Cancer pain and bleeding | No obvious systemic side effects, mild irritation when used locally, but good absorption | Y-National Pharmaceutical Standard Z14021231, Standard number: WS_3_-B-2752-97 | N |
| Delisheng injection | Beijing Zhengbang Pharmaceutical Co., Ltd. | *Red Ginseng*, *Astmgali Radix*, *Bufonis Venenum* (animal drug), *Mylabris* (animal drug) | *Panax ginseng C.A.Mey.;* *Astragalus mongholicus Bunge.* | Total solids, ＞20mg/mL; Anhydrous glucose, ＞3.5mg/mL;  Total amount of bufonis toxin and bufonisin, 12-18μg/mL; Cantharidin, 14-18μg/mL | Boost Qi and dissipate nodules | Cardiac stasis syndrome of primary liver cancer in middle and late stages, right abdomen mass, unabated pain, less abdominal distension, fatigue | Frequent urination, urgency, etc., urinary system irritation symptoms, occasionally hematuria and proteinuria can be seen; liver and kidney damage may occur, occasionally nausea and vomiting, abdominal distension. | Y-National Pharmaceutical Standard Z20010135, National Drug Standard WS_3_-134(Z-019)-2006(Z) | N |
| Huachansu injection | Anhui Huarun Jinchan Pharmaceutical Co., Ltd. | Toad skin (animal drug) | *-* | Serotonin, ＞0.50% (mg/ml) | Detoxification, swelling, pain relief | Middle and late stage tumors, chronic hepatitis B, etc. | Vascular stimulation; neutropenia; sinus bradycardia or tachycardia, chest tightness, palpitations, blood pressure changes; allergies, anaphylactic shock | Y-National Pharmaceutical Standard Z34020274, National executive standard WS_3_-B-3045-98 | N |
| Kangai injection | Changbaishan Pharmaceutical Co., Ltd. | *Astmgali Radix* 300g, *Ginseng Radix Et Rhizoma* 100g, Matrine 10g (Chemical medicine) | *Astragalus mongholicus Bunge.;* *Panax ginseng C.A.Mey.* | Total amount of ginsenoside Rgl and Re, ＞0.1mg/mL; Oxymatrine, 9.0-11.0mg/mL | Replenishing Qi and strengthening the body's immune function | Primary liver cancer, lung cancer, rectal cancer, malignant lymphoma and gynecological malignant tumors; leukopenia and hypoxia caused by various causes; chronic hepatitis B | Chills, fever, allergic-like reactions; rash, itching, flushing; nausea, vomiting, abdominal pain; chest tightness, palpitations, dizziness, headache, difficulty breathing, etc. | Y-National Pharmaceutical Standard Z20026868, Standard number: Ws-11222 (ZD-1222) -2002 | N |
| Kanglaite injection | Zhejiang Kanglaite Pharmaceutical Co., Ltd. | Coix seed oil for injection 100g, Soy lecithin for injection, Glycerin for injection, Water for Injection | *Coix lacryma-jobi L.* | Glycerol trioleate, ＞12.0mg/g | Replenishing Qi, nourishing yin, eliminating nodule | Primary non-small cell lung cancer and primary liver cancer, combined with radiotherapy and chemotherapy, have a certain synergistic effect, and have a certain anti-cachexia and analgesic effect on patients with advanced tumors | Occasionally, fat allergies, such as increased body temperature, mild nausea, and chills, most of the symptoms can disappear naturally after 3 to 5 days of use. Occasional mild phlebitis | Y-National Pharmaceutical Standard Z10970091, National Food and Drug Administration National Drug Standard WS3-301 (Z-038) -2006 (Z) -2013 | N |
| Shenfu injection | China Resources Sanjiu (Ya'an) Medical & Pharmaceutical CO., LTD.;  Ya'an Sanjiu Pharmaceutical Co., Ltd. | *Red Ginseng*, *Aconiti Lateralis Radix Praeparata* | *Panax ginseng C.A.Mey.;* *Aconitum carmichaeli Debeaux* | Aconitine, ＜0.1mg/mL; Ginsenoside Rb1, ＞0.5mg/mL | Go back to Yang, save the crisis, benefit Qi, and fix the fall | Syndrome of syncope of Yang Qi burst; palpitation, panting, cough, stomachache, diarrhea, arthralgia caused by Yang deficiency (Qi deficiency), etc. | Itching, facial flushing; nausea, vomiting, diarrhea; dizziness, headache, chills, high fever, difficulty breathing | Y-National Pharmaceutical Standard Z51020664, Z20043116, Z51021920, Z20043117; Standard number: WS3-B-3427-98 | N |
| Shenmai injection | Ya'an Sanjiu Pharmaceutical Co., Ltd.;  Zhengda Qingchunbao Pharmaceutical Co., Ltd.;  Hebei Shenwei Pharmaceutical Co., Ltd.;  Sichuan Chuanda West China Pharmaceutical Co., Ltd.;  Yunnan Gejiu Biopharmaceutical Co., Ltd.;  Sichuan Shenghe Pharmaceutical Co., Ltd.;  Dali Pharmaceutical Co., Ltd. | *Red Ginseng*, *Radix Ophiopogonis* | *Panax ginseng C.A.Mey.;* *Ophiopogon japonicus (Thunb.) Ker Gawl.* | Ginsenoside Re, 0.80-2.00mg/mL; Total amount of ginsenoside Rg1 and Re, ＞0.10mg/mL | Yiqi, fixed shedding, nourishing yin, nourishing body fluid, regenerating veins | Coronary heart virus myocarditis, chronic cor pulmonale and granulocytopenia can improve the immune function of cancer patients, enhance their immune function when combined with chemotherapeutic drugs, and reduce the toxic and side effects caused by chemotherapeutic drugs. | Allergies; systemic damage; shortness of breath, cough; arrhythmia, tachycardia, increased blood pressure; nausea, vomiting; dizziness, headache; rash, itching, phlebitis, lower back pain, etc. | Y-National Pharmaceutical Standard Z51021845, Z33020019, Z13020889, Z51021353, Z53021720, Z51021263, Z20093649; National Food and Drug Administration National Drug Standard WS3-B-3428-98-2010Z | N |
| Shenqifuzheng injection | Livzon Group Limin Pharmaceutical Factory | *Codonopsis Radix*, *Astmgali Radix* | *Codonopsis pilosula (Franch.) Nannf.*; *Astragalus mongholicus Bunge.* | Total solids, ＞13.0mg/mL; Astragaloside IV, 0.12-0.40mg/mL; Anhydrous glucose, ＞3.5mg/mL | Replenishing qi and supporting positive | Spleen-lung Qi deficiency causes fatigue, lack of Qi and laziness, and dizziness due to spontaneous sweating. Auxiliary treatment for lung cancer and gastric cancer with the above symptoms | Mild bleeding, low fever, stomatitis, drowsiness | Y-National Pharmaceutical Standard Z19990065, Standard Number WS3-387(Z-50)-2003(Z)-2011 | N |
| Shengmai injection | Changshu Leiyunshang Pharmaceutical Co., Ltd.;  Shanghai Hutchison Pharmaceutical Co., Ltd.;  Ya'an Sanjiu Pharmaceutical Co., Ltd.;  Jiangsu suzhong pharmaceutical group co. LTD.;  Sichuan Chuanda West China Pharmaceutical Co., Ltd.;  Wuliangye Group Yibin Pharmaceutical Co., Ltd.;  Shanxi Taihang Pharmaceutical Co., Ltd.;  Jilin Jian Yisheng Pharmaceutical Co., Ltd. | *Red Ginseng* 100g, *Radix Ophiopogonis* 312g, *Schisandrae Chinensis Fructus* 156g | *Panax ginseng C.A.Mey.;* *Ophiopogon japonicus (Thunb.) Ker Gawl.;* *Schisandra chinensis (Turcz.) Baill.* | Ginsenoside Rg1, ＞0.08mg/mL; Ginsenoside Re, ＞0.04mg/mL; Schisandrin A, ＞3μg/mL | Replenishing qi and nourishing yin, restoring pulse, fixing prolapse | Palpitation, shortness of breath, cold limbs, sweating, desperate pulse and myocardial infarction, cardiogenic shock, septic shock according to the above syndromes | Allergic reactions; systemic damage; rash, itching, sweating; digestive system damage; heart palpitations, increased blood pressure, arrhythmia, decreased blood pressure; damage to the mental and nervous system; respiratory system damage; phlebitis, local pain; Pain, myalgia, abnormal vision | Y-National Pharmaceutical Standard Z20044155, Z31020219, Z51021882, Z20053993, Z51021356, Z51022475, Z14020812, Z22025251; Executive Standard WS3-B-2865-98 | N |
| Xiaoaiping injection | Tonghua Jinma Pharmaceutical Group Co., Ltd.;  Nanjing Shenghe Pharmaceutical Co., Ltd. | *Marsdeniae Tenacissimae Caulis* Extract 200ml | *Marsdenia tenacissima (Roxb.) Moon* | Total solids, 35.0-45.0mg/mL; Chlorogenic acid, 9.0-13.0mg/mL | Clearing away heat and detoxification | Esophageal cancer, gastric cancer, lung cancer and liver cancer can be treated with adjuvant therapy of radiotherapy and chemotherapy. | Allergic reactions, anaphylactic shock; migratory muscle pain, joint pain; systemic reactions; rash, itching, sweating; digestive system damage; respiratory system damage; chest tightness, palpitations, increased or decreased blood pressure; dizziness, headache; injection Site pain, phlebitis | Y-National Pharmaceutical Standard Z20025869, Z20025868; Standard Number WS-10630-(ZD-0630)-2002 | N |
| Javanica oil emulsion injection | Guangzhou Baiyunshan Mingxing Pharmaceutical Co., Ltd.;  Jiangsu Jiuxu Pharmaceutical Co., Ltd.;  Pharmaceutical University of LeiYunShang Pharmaceutical Co. Ltd. | *Bruceae Fructus* oil 100mL | *Brucea javanica (L.) Merr.* | Oleic acid, 9.0-12.0% (g/g) | Anticancer drugs | Lung cancer, brain metastasis of lung cancer and digestive tract tumors | No obvious toxic and side effects, and occasional gastrointestinal discomfort such as greasy feeling, nausea and anorexia. | Y-National Pharmaceutical Standard Z44021325, Z19993152, Z21020639; Standard number: WS3-B-2739-97 | N |
